# FGFR1 and WT1 are markers of human prostate cancer progression

**DOI:** 10.1186/1471-2407-6-272

**Published:** 2006-11-30

**Authors:** Elizabeth Devilard, Franck Bladou, Olivier Ramuz, Gilles Karsenty, Jean-Philippe Dalès, Gwenaëlle Gravis, Catherine Nguyen, François Bertucci, Luc Xerri, Daniel Birnbaum

**Affiliations:** 1 Centre de Recherche en Cancérologie de Marseille, Département d'Oncologie Moléculaire, UMR599 Inserm et Institut Paoli-Calmettes, Marseille, France; 2 Département d'Urologie, Hôpital Salvator, Marseille, France; 3 Département de Biopathologie, Institut Paoli-Calmettes, Marseille, France; 4 Département de Pathologie, Hôpital Nord, Marseille, France; 5 Faculté de Médecine, Université de la Méditerranée, Marseille, France; 6 Département d'Oncologie Médicale, Institut Paoli-Calmettes, Marseille, France; 7 Laboratoire TAGC, ERM206, Marseille-Luminy, France

## Abstract

**Background:**

Androgen-independent prostate adenocarcinomas are responsible for about 6% of overall cancer deaths in men.

**Methods:**

We used DNA microarrays to identify genes related to the transition between androgen-dependent and androgen-independent stages in the LuCaP 23.1 xenograft model of prostate adenocarcinoma. The expression of the proteins encoded by these genes was then assessed by immunohistochemistry on tissue microarrays (TMA) including human prostate carcinoma samples issued from 85 patients who had undergone radical prostatectomy.

**Results:**

*FGFR1*, *TACC1 *and *WT1 *gene expression levels were associated with the androgen-independent stage in xenografts and human prostate carcinoma samples. MART1 protein expression was correlated with pT2 tumor stages.

**Conclusion:**

Our results suggest that each of these four genes may play a role, or at least reflect a stage of prostate carcinoma growth/development/progression.

## Background

Prostate adenocarcinoma is the most common cancer in men in western countries, and is responsible for about 6% of overall cancer deaths [1]. Localized prostate adenocarcinoma is usually treated by either surgery or radiotherapy. In the early stages, tumor growth is dependent on androgen stimulus and androgen ablation may be used as a complementary therapy. The tumor then progresses to an androgen-independent stage against which hormone therapy has no effect. Currently, there is no effective therapy against androgen-independent prostate cancer.

The molecular biology of prostate cancer is not well understood. Several previous reports have proposed candidate molecules linked to hereditary prostate cancer [3,4]. GSTP1, PTEN, TP53, and androgen receptor (AR) are mutated or deregulated in sporadic prostate cancer [5] and may become targets for innovative therapies. Recently, DNA microarrays experiments have identified other potential prognostic markers and/or targets, such as Hepsin/TMPRSS1, PSMA, and MMR genes [6,7] and gene fusions [8]. Overall, little is known about the progression from androgen-dependent to androgen-independent stages [2].

The LuCaP 23.1 human prostate carcinoma xenograft model [9] mimics the different stages of tumor growth and may be an adequate system to identify the molecular events associated with cancer progression [10]. Like for human samples, DNA microarray analyses of xenograft model systems have led to the discovery of several genes associated with cancer progression. However, although xenograft model systems are invaluable for gene discovery studies, as well as for experimental therapeutics, there is concern that growth of human cancer cells in an immunocompromised mouse host may not always be representative of progression of cancer in patients [11]. Combined gene and protein expression profilings – e.g. DNA microarrays and tissue microarrays (TMA) [12] – may allow easier or quicker validation of results provided by xenograft studies.

In the present work, we used DNA microarrays to identify candidate genes correlated with progression to androgen-resistant stage in the LuCaP 23.1 xenograft model. The expression of candidate genes with available and well-performing antibody was then assessed by immunohistochemistry (IHC) on TMA including human prostate carcinoma samples from 85 patients who had undergone radical prostatectomy.

## Methods

### Patient samples

Formalin-fixed, paraffin-embedded samples were obtained from 85 distinct prostatectomy specimens (Table 1). They included 80 tumor samples issued from prostatectomy specimens of carcinoma ranging from pT2aN0M0 to pT3bN0M0 stages (2002 pTNM classification of UICC), 5 carcinoma samples from carcinomas with distant metastases (N1 or M1 stages) classified as "hormone refractory" (HR), 11 benign prostate tissue samples, studied as control samples (these samples were obtained from benign tissue areas in 11 of the 85 above-mentioned prostatectomy specimens). This study was approved and executed in compliance with our institutional review board.

### LuCaP 23.1 xenograft model

Fresh frozen tumor samples were obtained from four different mice bearing the LuCaP 23.1 xenograft [9]. Each mouse was sampled three times, reflecting three distinct stages of tumor progression: primary tumor (day 1 post-transplantation), hormone sensitive (HS) tumor (day 7 post-transplantation), and hormone refractory (HR) tumor (day 114 post- transplantation).

### RNA extraction

Total RNA was extracted from tissue samples by lysis in guanidium isothiocyanate and centrifugation over a cesium chloride cushion following standard protocols [13]. RNA quality was assessed by denaturing formaldehyde agarose gel electrophoresis and reverse-transcribed PCR (RT-PCR) amplification of the β2-microglobulin transcript.

### DNA microarrays preparation and analysis

We used DNA microarrays to compare the mRNA expression profiles of ~1.000 selected genes between primary, HS, and HR tumors in the LuCaP 23.1 xenograft model of prostate carcinoma. RNA extracts were pooled according to the three distinct stages of tumor progression in the LuCaP 23.1 model (4 samples/tumor stage). DNA microarray hybridizations were done as previously described [14,15] on home-made nylon DNA microarrays (TAGC, Marseille-Luminy, France), which contained spotted PCR products from 945 human cDNA clones. Most genes were selected for a proven or putative implication in cancer and/or in immune reactions. Microarrays were hybridized with 33P-labeled probes made from 5 μg of total RNA. Probe preparations, hybridizations, and washes were done as previously described [14]. Briefly, 5 μg of total RNA were retrotranscribed in the presence of [-33P] dCTP (Amersham Biosciences). Hybridizations were done during 48 hours at 68°C in a final volume of 10 mL of buffer. After washes, arrays were exposed for 24 hours to phosphorimaging plates. Detection scanning was done with a FUJI BAS 5000 machine at 25-μm resolution (Raytest, Paris, France) and quantification of hybridization signals with the ArrayGauge software (Fuji Ltd, Tokyo, Japan). All hybridization images were inspected for artifacts, and aberrant spots or microarray regions were excluded from analyses. Data were analyzed as previously reported [18]. Hierarchical clustering was applied to the tissue samples and the genes using the *Cluster *program developed by Eisen [16].

All data are compliant with Minimum Information about Microarray Experiment (MIAME) guidelines and have been submitted to Gene Expression Omnibus (GEO) database [**GEO: GSE6284**].

### Tissue microarray (TMA) construction

The TMA included 96 formalin-fixed, paraffin-embedded human prostate tissue samples. TMA was prepared as described [17] with slight modifications. For each tumor, one representative tumor area was carefully selected from a hematoxylin- and eosin-stained section of a donor block. Core cylinders with a diameter of 1,2 mm each were punched from each of these areas and deposited into a recipient paraffin block using a specific arraying device (Beecher Instruments, Silver Spring, MD). Five-μm sections of the resulting microarray block were made and used for IHC analysis after transfer to glass slides.

### Immunohistochemical analysis

The antibodies used are listed in Table 2. PSA was used as a positive control of prostate tissue. Immunohistochemistry (IHC) was performed by using standard protocols. Briefly, IHC was performed on 5-μm sections of formalin-embedded tissue specimens. They were deparaffinized in histolemon (Carlo Erba Reagenti, Rodano, Italy) and rehydrated in graded alcohol. Antigen enhancement was done by incubating the sections in target retrieval solution (DAKO, Copenhagen, Denmark) as recommended. Slides were then transferred to a DAKO autostainer. Staining was done at room temperature as follows: after washes in phosphate buffer, followed by quenching of endogenous peroxidase activity by treatment with 0.1% H_2_O_2_, slides were first incubated with blocking serum (DAKO) for 10 min and then with the affinity-purified antibody for 1 hour. After washes, slides were incubated with biotinylated antibody against rabbit Ig for 20 min followed by streptavidin-conjugated peroxidase (DAKO LSABR2 kit). AEC was used as chromogen, according to the supplier's recommendations for the DAKO LSAB2 kit (Dakocytomation, Glosturp, Denmark). The tissue sections were then counterstained with haematoxylin and coverslipped using Aquatex mounting solution (Merck, Darmstadt, Germany). IHC staining was assessed by two pathologists (OR, LX) and classified into two categories: negative *versus *positive IHC staining. Each IHC staining was obtained and analyzed twice.

### RT-PCR analysis

RT-PCR was used to validate expression data on some of the samples included in the TMA. The primers and PCR amplification conditions for each tested gene are listed in Tables 3 and 4. Total RNA was extracted from 17 frozen tissue samples obtained from the prostatectomy specimens included in the TMA study. RNA extraction was done by standard protocols [13]. One μg of total RNA was reverse-transcribed at 42°C for 45 min, in a final volume of 20 μl containing: 1× reverse transcriptase buffer (Invitrogen Corp, Carlsbad, CA), 5 mmol/l MgCl_2 _(Invitrogen), 1 mmol/l dXTP (Invitrogen), 10 mmol/l dithiorthreitol (Invitrogen), 5 μmol/l random hexamers (Roche Diagnostics, Meylan, France), 20 U RNAse inhibitor (Promega Biosciences, Madison, WI), 200 U superscript reverse transcriptase (Invitrogen). Reverse transcriptase was inactivated by heating (99°C for 3 min) and cooling (4°C for 5 min). A 4 μl aliquot of this reverse transcription solution was used for PCR using 50 pmol of each specific primer, 10 mmol dNTP (Invitrogen), 1.5 mmol/l MgCl_2 _(Invitrogen), and 10 × PCR buffer (Invitrogen) in a total volume of 50 μl (see Table 2 for PCR amplification conditions). Next to the last PCR cycle, a final extension reaction was performed (72°C for 7 min). The integrity and amount of RNA available for preparation of all the cDNAs were assessed by amplification of the actin gene. Each PCR reaction included a negative control with H_2_O instead of cDNA, a positive control for each cDNA studied (cDNA from PC3 cell line, and LnCaP cell line for *WT1 *and *TACC1 *respectively, and a human metastatic melanoma sample for *MART1*), and a negative control of reverse transcription reaction. The cDNA amplification products were separated by electrophoresis on 1% agarose gel and stained by ethidium bromide staining. The cDNA was then classified as either not amplified (-), or weakly amplified (+), or strongly amplified (++).

## Results

### Overexpression of FGFR1, MART1, TACC1, and WT1 mRNAs in tumor progression in the LuCaP 23.1 carcinoma model

**Figure 1:**
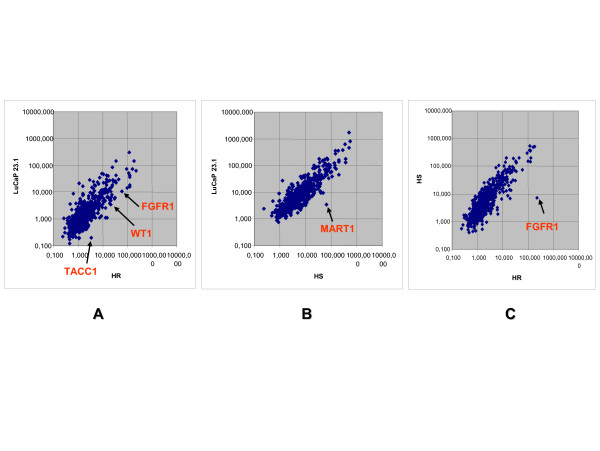
**Gene expression profiling of initial, HS, and HR stages of LuCaP 23.1 xenograft model of prostate carcinoma illustrated by scatter plots**. In each scatter plot, each point is a gene. **A: **Comparison of gene expression levels between initial (vertical axis) and HR (horizontal axis) stage carcinomas. *FGFR1*, *WT1*, and *TACC1 *are overexpressed in HR stage. **B: **Comparison of gene expression levels between initial (vertical axis) and HS (horizontal axis) stage carcinomas. *MART1 *is overexpressed in HS stage. **C: **Comparison of gene expression levels between HS (vertical axis) and HR (horizontal axis) stage carcinomas. As shown in A, *FGFR1 *is overexpressed in HR stage.

The respective expression levels of 945 genes in primary, HS, and HR stages of LuCaP 23.1 xenograft prostate carcinoma were compared. They are shown in Figure 1. A gene was considered as differentially expressed when the ratio between its median mRNA level in the primary LuCaP tumor sample and its mRNA level in the HR/HS tumor sample was more than 2-fold. This threshold was defined according to our previous reproducibility experiments [18]. Comparison between primary and HR stages showed that 27 genes were differentially expressed (Table 5). *FGFR1, TACC1 *and *WT1 *were overexpressed in HR LuCaP 23.1 carcinoma cells (Figure 1A). Comparison between primary and HS stages showed overexpression of 9 genes (Table 5), including *MART1*, in HS carcinoma cells (Figure 1B). Although most genes were expressed at the same level in HS and HR stages, *FGFR1 *was clearly overexpressed in HR stage (Figure 1C).

### FGFR1, TACC1 and WT1 proteins display high levels of expression in advanced stages of human prostate carcinoma

We then assessed the expression of four candidate proteins – FGFR1, MART1, TACC1 and WT1 – in human prostate carcinoma samples by IHC. This choice was supported by the availability of a corresponding antibody performing well on paraffin-embedded sections. To this purpose, we constructed a TMA that included 85 prostate carcinoma samples from 85 patients and 11 benign prostate tissue samples issued from 11 of these 85 patients.

**Figure 2:**
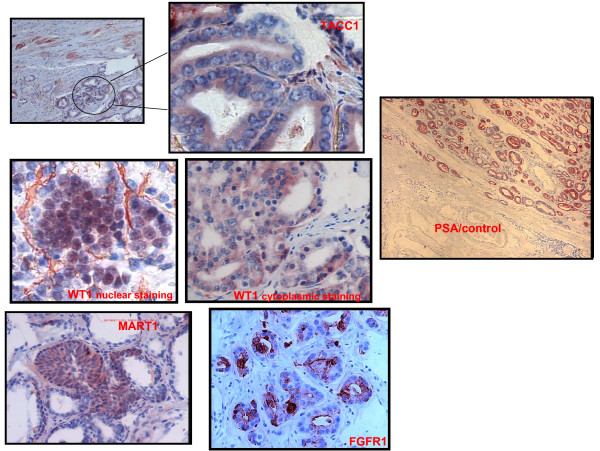
**Immunohistochemistry of FGFR1, TACC1, WT1, and MART1 protein expression in human prostate tissue samples deposited in tissue microarray**. **A: **FGFR1, MART1, and TACC1 IHC stainings are cytoplasmic, while WT1 staining is both nuclear and cytoplasmic in tumor cells. **B: **FGFR1, MART1, TACC1, and WT1, expression is more frequent in prostate carcinoma than in benign tissue samples. PSA is used as a positive control, which reacts with prostate secretory and ductal epithelium in normal and neoplastic tissue (demonstration of a neoplasm being of prostatic origin). In the table are indicated the percentages of positive samples with emphasis on a particular stage (in parenthesis).

Results are shown in Table 6 and Figure 2. FGFR1, MART1, and TACC1 IHC staining was cytoplasmic, while WT1 IHC staining was both nuclear and cytoplasmic (Figure 2). Benign prostate tissue expressed FGFR1 and WT1 less frequently than carcinoma samples. Among the 86 tumors, pT3 carcinoma samples expressed FGFR1 and WT1 proteins more frequently than pT2 samples. MART1 was expressed mainly in pT2 carcinomas. MART1 IHC expression was specific to malignant cells. The high expression of FGFR1, WT1 in pT3 tumors and MART1 in pT2 tumors was statistically significant using a chi2 test (Table 6).

### MART1, TACC1, and WT1 mRNA levels in human prostate samples correlate with IHC findings

**Figure 3:**
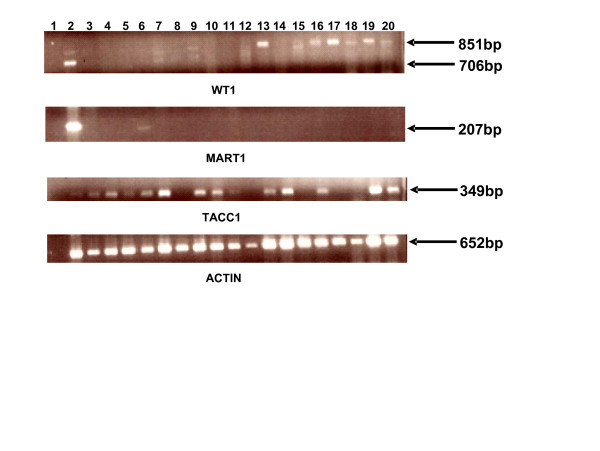
**RT-PCR assessment of *MART1, TACC1*, and *WT1 *mRNA expression in benign and neoplastic human prostate carcinoma samples**. - Lanes 1 and 2: negative control (with H_2_O instead of cDNA), a positive control for each cDNA studied (cDNA from PC3 cell line and from LNCAP cell line for *WT1 *and *TACC1 *respectively, and a human metastatic melanoma sample for *MART1*. - Lanes 3 and 4: n°1 paired benign and malignant (pT2) tissue samples. - Lanes 5 and 6: n°2 paired benign and malignant (pT2) tissue samples. - Lanes 7–10: distinct pT2 carcinoma samples. - Lanes 11–15: distinct pT3 carcinoma samples (13 and 15 are M+ carcinomas). - Lanes 16–20: distinct HR carcinomas samples. 1^st ^line: *WT1 *mRNA is detected as a 706 bp PCR product (variant) in 2/6 pT2 carcinoma samples, and as a 851 bp product (wild-type isoform) in 1/2 N1/M1 and 5/5 HR carcinoma samples. No *WT1 *mRNA is present in benign prostate tissue samples. 2^nd ^line: *MART1 *mRNA is expressed in only 1/6 pT2 carcinoma sample, but neither in any advanced (pT3 and/or N1/M1) stage nor HR carcinoma samples. 3^rd ^line: *TACC1 *mRNA is more strongly expressed in both pT2 and pT3 carcinoma than in benign tissue samples. *TACC1 *mRNA is also expressed in 3/5 HR samples. 4^th ^line: *α-actin *mRNA amplification is used as a quality control for mRNA extraction and RT-PCR reaction.

We used RT-PCR to assess whether *MART1*, *TACC1*, and *WT1 *mRNA levels were correlated with IHC expression levels in 2 benign and 16 malignant prostate samples. Because the correlation between FGFR1 transcriptional and post-transcriptional expression has been extensively discussed in the literature (19), we did not assess *FGFR1 *mRNA levels in our samples. Results are shown in Figure 3.

*MART1 *mRNA was weakly expressed in only 1/6 pT2 carcinoma, without any expression in pT3 carcinoma or HR (N1 and/or M1) samples (Figure 3). *TACC1 *mRNA expression was stronger in pT2 and pT3 carcinomas than in benign prostate tissue samples (Figure 3). Furthermore, *TACC1 *mRNA expression was strong in HR stage carcinoma samples (Figure 3). A *WT1 *mRNA variant (706 bp) was expressed in 2 of 6 pT2 carcinoma samples, while the major *WT1 *mRNA was strongly expressed in pT3 and HR (N1 and/or M1) carcinoma samples.

## Discussion

Using three methods of analysis (DNA microarrays, TMA, and RT-PCR), we have shown that FGFR1, TACC1 and WT1 have much higher levels of expression in human prostate carcinoma than in benign prostate tissue samples, at both mRNA and protein levels. We have also found that *FGFR1 *and *WT1 *mRNA are preferentially expressed in pT3 and/or N1/M1 carcinoma samples, and that *MART1 *expression is correlated with HS stage LuCaP 23.1 carcinoma and pT2 prostate carcinoma.

High-throughput screening techniques provide opportunities to identify new diagnostic or prognostic markers and innovative therapeutic targets in the whole field of oncology. TMA is a powerful tool to validate DNA microarrays data and extend the scope of gene expression profiling to the post-transcriptional level. Several previous studies [17,20,22,23] have emphasized how TMAs are useful to validate the use of candidate prostate carcinoma markers in routine (IHC) conditions [21].

### Potential new markers for prostate cancer progression

We found that expression of FGFR1, WT1, and, to a lesser extent, TACC1 protein is upregulated in advanced stages (pT3 and/or N1/M1) of prostate cancer, whereas MART1 is mainly expressed in localized (pT2) stage prostate cancer. In the same way, we observed that the corresponding mRNAs – *FGFR1, TACC1*, and *WT1 *on the one hand, and *MART1 *on the other hand – are overexpressed in HR and HS stage LuCaP 23.1 prostate carcinoma, respectively.

*FGFR1 *codes for a tyrosine kinase receptor for members of the FGF family of growth factors. It is a potential oncogene, amplified in breast cancers and rearranged in hematopoietic diseases. The case of FGFR1 in prostate cancer is rather clear and our results are in perfect agreement with previous data. Expression of FGFR1 is associated with increased proliferation and aggressive behavior of prostate cancer [24,25].

The *MelanA/MART1 *gene encodes a tyrosinase that is a marker of melanocytic differentiation [26]. It can be recognized by cytotoxic T cells [28] and has been considered as a target for immunotherapy [28]. A previous study has shown that the protein is expressed in lymph nodes from breast cancer patients [27]. Our results showed mRNA overexpression in HS stage LuCaP 23.1 model and IHC expression of MART1 in about 20% of prostate carcinomas, mainly pT2 stages. MART1 expression was strictly restricted to carcinoma cells, without any staining in benign prostate tissue. Therefore, we suggest that MART1 may be a marker of some intermediate, hormone sensitive stage of prostate carcinoma, and that its transient expression might be shut down during cancer progression towards hormone resistant and/or advanced clinical stages (T3 and/or N+ and/or M+).

TACC1 belongs to the TACC/taxins (Transforming Acidic Coiled-Coil) protein family. Taxins are centrosome and spindle-associated proteins involved in cell division [29]. Mammalian Taxins are probably involved in oncogenesis in different ways [29,30]. TACC1 maps to 8p11, a region that is amplified and rearranged in many malignancies [29-31]. We observed upregulation of TACC1 expression in prostate carcinoma. This is in agreement with previous reports focusing on other tumors [32]. Since we found TACC1 underexpressed in the majority of breast carcinoma [34], TACC1 role remains to be determined. As previously suggested, TACC1 might be involved in multiple complexes that may be deregulated in malignant conditions [33].

The *WT1 *(Wilms tumor 1) gene encodes a zinc finger transcription factor that modulates the expression of several genes encoding growth factors and receptors (epidermal growth factor receptor [35], insulin-like growth factor II [36], IGF1 receptor [36] and AR [37]. In Wilms tumor, different point mutations have been described in the *WT1 *locus, suggesting that WT1 altered protein may be directly involved in tumor formation. High WT1 expression levels have been reported in several malignancies [38-41], and have been linked to a poor prognosis [42]. WT1 expression and multidrug resistance are associated in some hematological malignancies, suggesting that WT1 may be a marker for chemoresistance [43]. We found high expression levels of WT1 in pT3 stage carcinoma samples, and expression of wild-type WT1 mRNA in both advanced stages (≥ pT3) and HR stage LuCaP 23.1 carcinomas. These results suggest that WT1 expression in prostate carcinoma may be associated with progression towards hormone resistance and that WT1 may be considered as a potential hormone resistance and prognostic marker in human prostate carcinoma. These hypotheses are supported by the strong repression of AR promoter by WT1 [44].

## Conclusion

**Figure 4:**
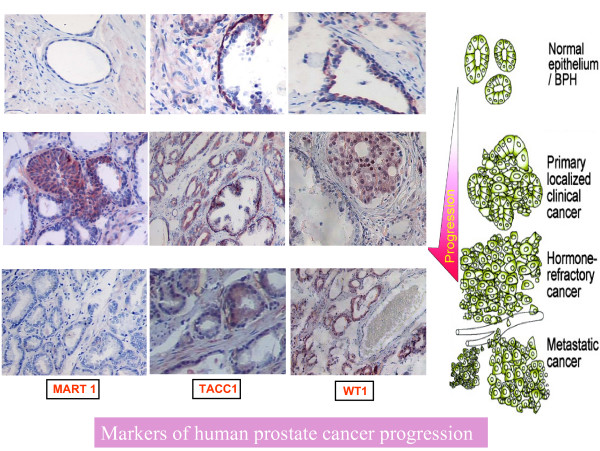
**Schematic representation of the conclusions**. Potential markers of prostate cancer progression, MART1, TACC1 and WT1, are shown expressed in representative sections of various stages.

In this study, we identified four candidate genes – *FGFR1*, *MART1, TACC1 and WT1 *– by gene expression profiling of different stages of prostate carcinoma in an animal model. The clinical relevance of these candidate genes was assessed by IHC on TMA of human prostate carcinoma samples. Our results suggest that each of these four genes may play a role, or at least reflect a stage of prostate carcinoma growth/development/progression. MART1 might be linked to hormone-sensitive growth and localized tumors, whereas FGFR1, TACC1 and WT1 could be associated with hormone-independent growth and advanced prostate carcinoma (see in Figure 4).

## Competing interests

The author(s) declare that they have no competing interests.

## Authors' contributions

ED carried out the micro array, tissue array and RT-PCR experiments, designed the study and drafted the manuscript.

FBl, GK, GG and JPD were involved in the acquisition of prostate tumor samples and clinical data.

OR, LX analyzed the immunohistochemistry results.

CN provided home made DNA microarrays.

FBe analyzed gene expression profiling data and helped to draft the manuscript.

DB and LX supervised the study. DB revised the manuscript and gave final approval of the version to be published.

All authors read and approved the final manuscript.

## Aknowledgements

We thank F. Birg and D. Maraninchi for encouragements. This work was supported by Institut Paoli-Calmettes and INSERM.

## Pre-publication history

The pre-publication history for this paper can be accessed here:



## Figures and Tables

**Table 1:** Patients and tumor characteristics.

Characteristics	All patients (n = 85)
Median age (years)	62
TNM classification:	
pT0	11
pT1	0
pT2a	10
pT2b	30
pT3a	28
pT3b	17
T3N1M1b	3
T3N1M1c	2
Gleason grading:	
5	4
6	8
7	53
8	15
9	5
Median follow-up (months)	60
Metastatic relapse (n)	4
Death (n)	3

**Table 2:** Antibodies and antigen retrieval procedures used for IHC

Protein	Origin	Clone	Dilution	Antigen Retrieval
FGFR1	SantaCruz	C15	1/200	Demask (DAKO)
MART1	DAKO	A103	1/2	Demask (DAKO)
TACC1	Upstate Biotechn.	07–229	1/500	Demask (DAKO)
WT1	Oncogene	WLM04	1/100	Citrate (DAKO)
PSA	DAKO	N1517	rtu*	*

*rtu: ready-to-use solution.

**Table 3:** Primers used for mRNA amplification by RT-PCR.

TACC1	F: AAATACGAAGAGACCCGGC
	R: TGTCCAGTTTCTCTTCTGCG
	
WT1	F: ATGAGGATCCCATGGGCCAGCA
	R: CCTGGGACACTGAACGGTCCCCGA
	
MART1	F: TGACCCTACAAGATGCCAAG
	R: TCAGCATGTCTCAGGTGTCT
	
Actin	F: AGCAAGAGAGGCATCCTGACC
	R: CTTCATGATGGAGTTGAAGGTAG

DNA sequences are listed in a 5'→3' orientation (F and R are the forward and reverse DNA strands, respectively).

**Table 4:** PCR conditions for RT-PCR amplification

GENE	N° of cycles	Denaturation	Annealing	Extension
WT1	35	94°C/30 sec	64°C/30 sec	72°C/30 sec
MART1	30	94°C/1 min	60°C/1 min	72°C/2 min
TACC1	30	94°C/1 min	55°C/1 min	72°C/2 min
ACTIN	25	94°C/30 sec	55°C/30 sec	72°C/30 sec

**Table 5:** 27 and 9 genes differentially expressed between primary and HR, and between primary and HS LuCaP 23.1 xenografts.

**HR vs LUCAP 23.1**	**Description**
*FGFR1*	Fibroblast growth factor receptor 1
*KRT19*	Cytokeratin 19
*CDK4*	Cyclin-dependent kinase 4
*AMFR*	Autocrine motility factor receptor
VEGFR1	Vegf receptor 1/FLT1
ITGA3	Integrin alpha 3/CD49C
MUC1	Polymorphic epithelial mucin
GRB7	GRB7 adaptor
RELA	NFKb, p65 subunit
BCL3	BCL3 protein
SRF	Serum response factor
MAX	MAX transcription regulator
NEO1	Neogenin homolog 1
*ITGB5*	Integrin beta 5
RXRA	Retinoic X receptor alpha
IGFBP4	Insulin-like growth factor binding protein
CASP9	Caspase 9
CREBBP	CREB binding protein
MMP15	Membrane-type matrix metalloproteinase 2
SMAD9	MAD homolog 9
BAK	Apoptosis regulator bak
MMP9	92 kDa gelatinase, matrix metalloproteinase-9
GATA1	GATA-binding protein 1
MRP5	ATP-binding cassette, sub-family C, member 5
PPP2R2C	PP2A BR gamma
EPOR	Erythropoietin receptor
FZD5	Frizzled 5, WNT pathway
**HS vs LuCaP 23.1**	
MART1	Melan-a protein
CSTB	Cystatin b
NR1H3	Nuclear receptor liver X receptor
VINC	Vinculin
STMN1	Stathmin, phosphoprotein p19
STAT1	signal transducer and activator of transcription 1
CBLB	Cas-Br-M ectropic retroviral transforming sequence b
WNT2	WNT factor 2
CDH12	Cadherin 12

**Table 6:** Expression of FGFR1, MART1, TACC1 and WT1 proteins in human prostate cancer.

**Factors**	**benign**	**pT2**	**pT3**	***p*-value**
	No. of patients (%)			

**Age, years**				1
*<=50*	0	0	0	
*>50*	11	39	46	
**Gleason**				**0,057**
*5*	0	3	1	
*6*	0	7	4	
*7*	0	25	28	
*8*	0	3	10	
*9*	0	0	4	
**FGFR1 status**				**0.001**
*negative*	9	27	17	
*positive*	2	12	29	
**MART1 status**				**0.029**
*negative*	11	30	43	
*positive*	0	9	3	
**TACC1 status**				**0.756**
*negative*	3	13	12	
*positive*	8	26	34	
**WT1 status**				**0.008**
*negative*	9	24	17	
*positive*	2	15	29	
